# Combined Signature of the Fecal Microbiome and Metabolome in Patients with Gout

**DOI:** 10.3389/fmicb.2017.00268

**Published:** 2017-02-21

**Authors:** Tiejuan Shao, Li Shao, Haichang Li, Zhijun Xie, Zhixing He, Chengping Wen

**Affiliations:** ^1^College of Basic Medical Science, Zhejiang Chinese Medical UniversityHangzhou, China; ^2^State Key Laboratory for Diagnosis and Treatment of Infectious Diseases, Collaborative Innovation Center for Diagnosis and Treatment of Infectious Diseases, The First Affiliated Hospital, College of Medicine, Zhejiang UniversityHangzhou, China

**Keywords:** gout, microbiome, metabolome, fecal, signature

## Abstract

This study employed microbiome and metabolome analysis to explore the fecal signatures of gout patients. Fecal samples from 52 male individuals (26 healthy controls and 26 gout patients) were analyzed by ^1^H NMR spectroscopy and Illumina Miseq sequencing. The signatures of microbiome showed being up-regulation of opportunistic pathogens, such as *Bacteroides, Porphyromonadaceae Rhodococcus*, *Erysipelatoclostridium* and *Anaerolineaceae*. The signatures of metabolome were some altered metabolites which may involve uric acid excretion, purine metabolism, and inflammatory responses. Meanwhile, the correlation between discrepant metabolites and microbial taxa indicated that they could be the combined signatures of gout. This study suggests that the combined analysis of the fecal microbiome and metabolome may effectively characterize diseases.

## Introduction

Human feces are complex and comprised of multiple elements, including proteins, nucleic acids, metabolites, human cells, and microorganisms. Disorder of any element can be related with the development of certain diseases. Hence, different human diseases are associated with different fecal levels of biomarkers. Over the past years, biomarkers of fecal material are gaining increased attention because they provide a non-invasive method for disease diagnosis. Fecal biomarkers could be microRNAs (Wu et al., 2014), proteins (Buderus et al., 2015), microorganisms (Narayanan et al., 2014), and metabolites (Ahmed et al., 2016). Quantification of the above biomarkers between disease and health may also clarify potential pathways of the pathogenesis of diseases.

The microbiota in the intestinal tract may be a significant fecal biomarker due to its crucial role in intestinal health, such as the digestion of food, protection of mucosal surfaces, and crosstalk with the host immune system (Hooper et al., 2012; Nicholson et al., 2012). The dysbiosis signatures of the fecal microbiota are associated with diseases such as obesity (Sanz et al., 2010), cancer (Zackular et al., 2014), inflammatory bowel disease (Scher et al., 2015), and ankylosing spondylitis (Costello et al., 2015). Generally, intestinal dysbiosis can cause fecal metabolites variation because most of the metabolites are derived from the microbiota (Lee and Hase, 2014), such as short-chain fatty acids (SCFA), trimethylamine, and amino acids (Smith et al., 2013; Falony et al., 2015). The fecal microbiome and metabolome are simultaneously found to be disordered in colorectal cancer or systemic lupus erythematosus patients compared to healthy controls (Weir et al., 2013; Hevia et al., 2014; Rojo et al., 2015). Therefore, the combined fecal microbiome and metabolome signature may provide reliable and comprehensive information to uncover fecal biomarkers of diseases, especially metabolic diseases.

Gout is a purine metabolic disorder disease that is characterized by elevated serum uric acid (UA) and deposition of urate in and around the joints. Gout patients suffer from arthritis attacks, severe pain, and degeneration of the first metatarsophalangeal joint (Rafey et al., 2003). The excretion of UA plays a significant role in the alleviation of pain. Healthy humans excrete UA through the kidneys and intestine. Therefore, metabolites in the intestine may be altered because of UA excretion. In addition, the intestinal microbiota can participate in the metabolism of purine and UA, such as *Escherichia coli* (Crane et al., 2013), *Lactobacillus*, and *Pseudomonas* (Hsieh et al., 2014). In conclusion, the intestinal fecal signature may be related with metabolites and microbiota in gout patients.

To explore the fecal signature of gout, ^1^H NMR and Illumina Miseq were employed to investigate the metabolic profile and microbial community of fecal extracts from gout patients and healthy individuals, respectively. The overall goal of this study was to ascertain if the combined signatures of microbiome and metabolome could characterize male gout patients.

## Materials and Methods

### Patient Population

The study protocol was approved by the Ethics Committee of Zhejiang Chinese Medical University, and written informed consent was obtained from each participant. A total of 26 male patients with diagnosed gout were recruited from three hospitals (Zhejiang Provincial Hospital of TCM, The Second Affiliated Hospital of Zhejiang Chinese Medical University, and Zhejiang Province People Hospital). All patients had suffered from gout for at least 12 months and did not receive any medical treatment within 1 month of study participation. The patients with comorbid disorders were excluded. The clinical diagnosis and blood examination reports of all patients were obtained from the hospitals. Twenty-six male volunteers were recruited by a routine physical examination. The healthy controls had no gastrointestinal tract disorders and did not receive antibiotics within 1 month of this study. In addition, there were no significant differences among the two groups in terms of age, smoking history, alcohol intake or dietary intake. The clinical data such as UA, body mass index (BMI), erythrocyte sedimentation rate (ESR), blood urea nitrogen (BUN) and serum creatinine (Cr) were shown in **Table 1**.

**Table 1:** Demographic and clinical chemistry characteristics of human subjects.

Characteristics	Gout patients	Healthy controls	*p*-value	FDR
Age mean ± *SD* [min, max]	43.60 ± 1.98 [25, 66]	39.42 ± 2.33 [20, 67]	0.285	0.2850
BMI (kg/m^2^) mean ± *SD* [min, max]	24.28 ± 0.31 [21.30, 27.16]	23.56 ± 0.23 [21.45, 25.89]	0.021	0.0315
ESR (mm/h) ± SD [min, max]	11.21 ± 1.72 [2, 41]	7.04 ± 0.54 [3, 16]	0.020	0.0315
UA (μmol/L) ± SD [min, max]	439.09 ± 13.56 [307, 569]	284.50 ± 11.05 [193, 409]	0.000	0.0000
BUN (mmol/L) ± SD [min, max]	6.21 ± 0.33 [3.25, 8.60]	4.98 ± 0.26 [2.9, 7.3]	0.000	0.0000
Cr (μmol/L) ± SD [min, max]	70.15 ± 4.11 [40.6, 104.3]	64.18 ± 4.35 [32.9, 129.3]	0.193	0.2316

### 16S rRNA Gene Tag Sequencing

Total DNA was extracted from thawed fecal samples using the QIAamp^®^ DNA Stool Mini Kit (Qiagen, Hilden, Germany) according to the manufacturer’s protocols. The extracted products were determined by agarose gel electrophoresis (1% w/v agarose). Quantification of the DNA yield was carried out using a NanoDrop2000 spectrophotometer (Thermo Scientific). The DNA was stored at -20°C for Illumina Miseq sequencing analysis.

The V3–V4 region of the 16S rRNA genes was amplified from the diluted DNA extracts with the primers 319f (5′-ACTCCTACGGGAGGCAGCAG-3′) and 806r (5′-GGACTACH VGGGTWTCTAAT-3′). PCR amplification was then performed in a 30 μl mixture containing 0.5 μl of DMSO, 1.0 μl of forward primer (10 mM), 1.0 μl of reverse primer (10 mM), 5.0 μl of DNA sample, 7.5 μl of ddH2O and 15.0 μl of Phusion High-Fidelity PCR Master Mix with HF Buffer (NEB). The reactions were hot-started at 98°C for 30 s, followed by 30 cycles of 98°C for 15 s, 58°C for 15 s, and 72°C for 15 s, with a final extension step at 72°C for 1 min. The PCR products were purified using an agarose gel DNA purification kit (Qiagen, Chatsworth, CA, USA). The amplicon library was prepared using a TruSeq^TM^ DNA sample preparation kit (Illumina Inc, San Diego, CA, USA). The sequencing reaction was conducted using Illumina MiSeq sequencing (2 × 300 bp; Hangzhou Guhe Information and Technology Co., Ltd., Zhejiang, China).

### ^1^H NMR Spectroscopy Measurements

Thawed fecal samples were suspended with Na^+^/K^+^ phosphate buffer (K_2_HPO_4_–NaH_2_-PO_4_, 0.1 M, pH 7.4) containing 100% D_2_O and 0.005% sodium 3-trimethylsilyl [2,2,3,3-d4] propionate (TSP). The suspension was subjected to freeze-thaw treatment (3 times) with liquid nitrogen and 20 × ultrasonication cycles (20 s vortex-10 s waiting) followed by centrifugation (12000 rpm, 4°C, 15 min). The supernatants were removed, filtered through 0.2 μm membrane filters, and 550 μL of each filtrate was transferred to 5 mm NMR tubes (Norell ST50-7, USA) for NMR spectroscopic analysis.

High resolution ^1^H NMR spectra were recorded on a Bruker Avance III 600 MHz spectrometer (Bruker, Biospin, Germany) using an inverse detection cryogenic probe. The sample temperature was controlled at 298 K. One-dimensional NMR spectra were recorded using a first increment of NOESY pulse sequence (recycle delay –90°–t1–90°–tm–90°–acquisition), and water suppression was achieved with weak continuous-wave irradiation during the recycle delay (2 s) and the mixing time (100 ms). The 90° pulse length was adjusted to approximately 10 μs, and 64 transients were collected into 32 k data points with a spectral width of 20 ppm.

The spectra were transformed with 1 Hz line broadening and zero filling, manually phased, and baseline corrected using the TOPSPIN 2.0 software. Metabolites were identified using information found in the literature (Saric et al., 2007; Le Gall et al., 2011) or on the web (Human Metabolome Database)^1^ and by use of the 2D-NMR methods including ^1^H–^1^H correlation spectroscopy (COSY), ^1^H–^1^H total correlation spectroscopy (TOCSY), ^1^H–^13^C heteronuclear multiple-bond correlation (HMBC), and ^1^H–^13^C heteronuclear single quantum correlation (HSQC) spectra.

### Statistical Analysis

Paired-end reads of sequencing were first merged and demultiplexed into patient samples using the Quantitative Insights Into Microbial Ecology (QIIME version 1.9; Caporaso et al., 2010). Before assembly, sequence reads were first filtered to remove low-quality or ambiguous reads, including reads lacking exact matching with the primer, reads containing ambiguous character (N), and reads with an average quality score <25. Only two reads with a sequence overlap longer than 20 bp were assembled. The assembled sequence reads with <400 bp or >500 bp were discarded. High-quality sequences were binned into 16S rRNA Operational Taxonomic Units (OTUs) and defined at ≥97% sequence homology. Chimera detection and removal were assessed using the GOLD reference database and uchime (Edgar et al., 2011). The taxonomic affiliation of each OTU was performed with QIIME against the SILVA database (Quast et al., 2013). Alpha diversity (Chao1, observed species, Simpson, Shannon, singles, doubles) was analyzed based on the OTUs table rarefied to 1000 reads, and 20,000 reads were finally extracted from each sample for the other analyses.

To test whether gut microbial species could be differentiated between gout patients and healthy controls, a metric multidimensional scaling method based on projection known as principal coordinates analysis (PCoA) was used. Each sample was mapped based on the overall microbial composition and assessed for similarities. The online software LefSe (Segata et al., 2011) was utilized to select and demonstrate differentially abundant taxonomy based on Kruskal–Wallis test and LDA (linear discriminant analysis) score.

The statistical processing of ^1^H NMR data was conducted as previously described (Shao et al., 2016). After manual corrections for phase and baseline distortion (Bruker BioSpin), ^1^H NMR spectra were referenced to the TSP signal (δ 0.0) and spectral regions 9.50–0.6 were integrated into regions with an equal width of 0.004 ppm using the AMIX software package (V3.8, Bruker BioSpin). The region containing the water resonance (δ 5.16–4.68) was removed. Each bucket was normalized to the total sum of the spectral integrals, and then the peak areas of identified metabolites were extracted to calculate metabolite percentage concentrations. Total metabolite concentrations were performed with multivariate data analysis using the SIMCA-P^+^ software package (version 11.0, Umetrics, Sweden). In this study, partial least squares discriminant analysis (PLS-DA) was performed to attempt to maximize the separation between classified groups of observations. The validity of the model was assessed with the R^2^X and Q^2^ values, reflecting the explained variables and the predictability of the model, respectively. The validity of the model was further evaluated with rigorous permutation tests (*n* = 200). Identified metabolite concentrations were calculated according to ^1^H chemical shifts. Metabolite concentrations within groups were compared using Mann–Whitney non-parametric test in SPSS software 16.0.

The correlation network was performed with a Spearman’s rank correlation coefficient on the discrepant taxonomy and metabolites in R program, and only connections with a *p*-value less than 0.01 were retained. Meanwhile, to correct the results of this, R-p-adjust method in *R* program were used to calculate FDR values.

## Results

### Intestinal Dysbiosis in Gout Patients

Fifty-two male individuals (26 healthy controls and 26 gout patients) were enrolled to study microbial profiles of stool samples. The results of Illumina Miseq sequencing shown each sample had at least 20,000 valid reads for OTU analysis. The intestinal microbiota of patients with gout was significantly different compared to healthy control subjects. The α diversity indices (Chao1, Observed species, Simpson, Shannon, Singles and Doubles) of the intestinal microbiota from the gout group were less than the healthy group (Supplementary Figure S1), suggesting that gout was associated with lower microbial diversity. Additionally, three-dimensional PCoA showed separation between the two groups, indicating that gout was the primary factor influencing the differences (Supplementary Figure S2).

**FIGURE 1:**
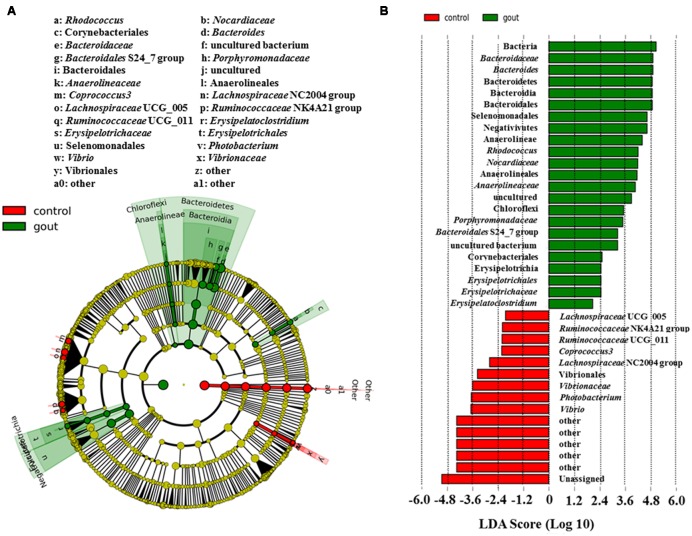
**LefSe identified the most differential genera between gout patients and healthy controls. (A)** Cladogram; **(B)** Histograms. Red and green represent healthy controls and gout samples, respectively.

**Figure 1** showed the discrepant microbial species with a reduced significance threshold (LDA score >2) between the two groups. The LefSe method revealed that the phylum Bacteroidetes and its derivative (Bacteroidia, Bacteroidales, *Bacteroidaceae* as well as *Bacteroidales* S24_7 group and *Porphyromonadaceae*), the phylum Chloroflexi and its derivatives (Anaerolineae, Anaerolineales, and *Anaerolineaceae*), the order *Corynebacteriales* and its derivative (*Nocardiaceae* and *Rhodococcus*), the class Erysipelotrichia and its derivatives (Erysipelotrichales, *Erysipelotrichaceae* and *Erysipelatoclostridium*), and the class Negativicutes and its derivative (Selenomonadales) were all higher in the intestinal microbiota from the gout patients. Conversely, the family *Vibrionaceae* and its derivatives (*Photobacterium* and *Vibrio*), the genus *Coprococcus* 3, *Lachnospiraceae* NC2004 group, *Lachnospiraceae* UCG_005, *Ruminococcaceae* NK4A214 group and *Ruminococcaceae* UCG_011 were all lower in the intestinal microbiota from the gout patients.

### Discrepancy of Metabolites in Gout Patients

**FIGURE 2:**
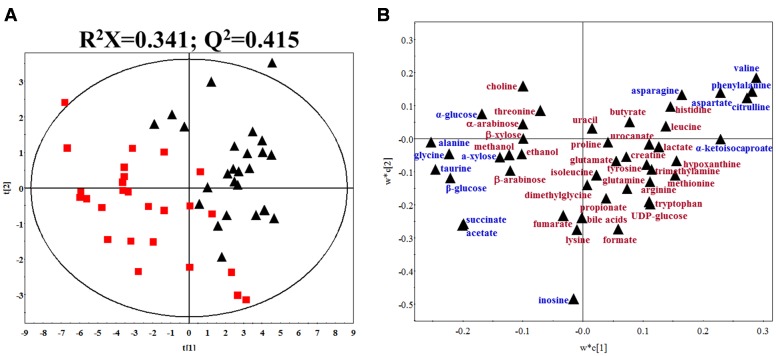
**(A)** PLS-DA score plot of gout patients and healthy controls based on the fecal metabolic profiles. The red box and black triangle represent gout and healthy controls samples, respectively. **(B)** PLS-DA loading plot. The blue font denotes the metabolites with VIP > 1.0, and the dark red font denotes the metabolites with VIP < 1.0.

Typical ^1^H NMR spectra of fecal extracts of gout and healthy controls were shown in Supplementary Figure S3. The spectra of fecal extracts showed 46 targeted metabolites in this study. The detailed information was provided in Supplementary Table S1. To maximize class discrimination and discover the gout-related fecal metabolites, a comparative PLS-DA model was utilized on the targeted metabolite data from the gout patients and healthy controls. The values for R^2^X and Q^2^ and the results of permutation tests indicated that the two models were of reasonable quality (**Figure 2A**). **Figures 2A,B** show the PLS-DA score plots and corresponding loading plots for human fecal extracts, respectively. Clear separations in the PLS-DA score plot based on 46 targeted metabolites were observed between the gout patients and healthy controls (**Figure 2A**). Differential metabolites were defined based on the variable importance for project values (VIP) value of PLS-DA. Metabolites with a VIP value greater than 1.0 were dispersed from the origin of the loading plot and were considered as the primary contributors for classification of the groups (**Figure 2B**).

According to the PLS-DA model analysis, 15 targeted metabolites with a VIP above 1.0 were selected and subjected to a significance test with Mann–Whitney non-parametric test. As shown in **Table 2**, 14 of 15 metabolites were significantly different with *p* < 0.05. Compared with healthy controls, gout patients had significantly higher concentrations of alanine, glycine, taurine, succinate, acetate, α-glucose, β-glucose and α-xylose, and significantly lower concentrations of valine, asparagine, aspartate, citrulline, phenylalanine and α-ketoisocaproate. The detailed concentrations of the 14 significant metabolites were shown in Supplementary Figure S4.

**Table 2:** Summary of the identified differential metabolites between gout patients and healthy controls (VIP > 1).

Metabolites	δ (ppm)	VIP	*p*-value	FDR
Valine	2.27 (m)	1.76	0.000	0.0000
Phenylalanine	7.38 (m)	1.70	0.000	0.0000
Citrulline	1.87 (m)	1.64	0.000	0.0000
Inosine	8.34 (s)	1.55	0.076	0.0760
Alanine	3.79 (q)	1.52	0.000	0.0000
Taurine	3.43 (t)	1.47	0.000	0.0000
Aspartate	2.82 (m)	1.40	0.000	0.0000
Acetate	1.92 (s)	1.39	0.001	0.0014
α-ketoisocaproate	2.61(d)	1.38	0.000	0.0000
Succinate	2.41 (s)	1.37	0.001	0.0014
β-glucose	3.50 (t)	1.34	0.000	0.0000
Glycine	4.65 (s)	1.33	0.000	0.0000
α-glucose	3.71 (t)	1.07	0.003	0.0035
Asparagine	2.96 (dd)	1.02	0.002	0.0025
α-xylose	2.14 (m)	1.02	0.021	0.0225

### Correlations of Discrepant Microbial Taxa and Fecal Metabolites

**FIGURE 3:**
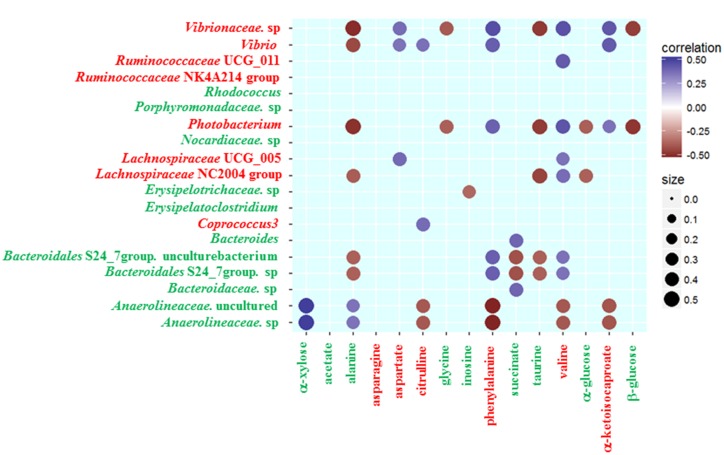
**Heatmap of Spearman’s rank correlation coefficients of the relative abundances of different gut microbiota at the family and genera level and fecal metabolites in healthy controls and gout patients.** Circle sizes and color intensity represent the magnitude of correlation. Blue circles = positive correlations; brown circles = negative correlations. Red text denotes depletion in gout patients; green text denotes enrichment in gout patients.

To further analyze the associations of alterations in fecal metabolome and microbiome, we conducted the correlation of 19 discrepant microbial taxa at the family and genera level and 15 discrepant metabolites using the Spearman’s rank correlation method. As shown in **Figure 3**, no associations were involved in some microbial taxa (*Ruminococcaceae* NK4A214 group, *Rhodococcus*, *Porphyromonadaceae* sp., *Nocardiaceae* sp. and *Erysipelatoclostridium*) and some fecal metabolites (acetate, asparagine). However, the associations were observed among most fecal signatures of metabolome and microbiome.

Some gout-enriched microbial taxa were associated with the gout-enriched metabolites. The positive associations were observed between *Bacteroides* and succinate, between *Anaerolineaceae* and alanine or α-xylose. The negative associations were observed between *Erysipelotrichaceae* sp. and inosine, between *Bacteroidales* S24_7 group and alanine or succinate or taurine. Meanwhile, some gout-enriched microbial taxa were also associated with the gout-depleted metabolites. The positive associations were observed between *Bacteroidales* S24_7 group and valine or phenylalanine.

The gout-depleted microbial taxa were negatively associated with the gout-enriched metabolites. For example, *Photobacterium* was negatively associated with alanine, glycine, taurine, α-glucose and β-glucose. However, the gout-depleted microbial taxa were positively associated with the gout-depleted metabolites. For instance, the positive associations were observed between *Coprococcus 3* or *Vibrio* and citrulline.

Although the correlation between metabolites and microbial taxa doesn’t mean that they exhibit any biological interaction, they have the potential to be a combination of fecal signatures.

## Discussion

Accumulating evidence indicates that gut microbiota interact with gout, which is a concerning strategy for characterizing and treating gout patients (Kim et al., 2014; Guo et al., 2016). We used high-throughput sequencing of the V3–V4 region of the 16S rRNA gene to characterize the fecal microbiome, ^1^H NMR spectra assaying of small molecules to characterize the fecal metabolome. To the best of our knowledge, this is the first attempt to combine the signatures of gout by integrating the microbiome and metabolome.

Some alterations of the gut microbiome have the potential to distinguish gout patients from healthy controls. In gout, *Bacteroides caccae* and *Bacteroides xylanisolvens* are enriched yet *Faecalibacterium prausnitzii* and *Bifidobacterium pseudocatenulatum* depleted (Guo et al., 2016). Our study also finds the family *Bacteroidaceae* sp. and its genera *Bacteroides* enriching in male gout patients. Meanwhile, the enrichment of genera *Bacteroides* are associated with other autoimmune diseases, such as rheumatoid arthritis (Zhang et al., 2015), systemic lupus erythematosus (Hevia et al., 2014) and diabetes (Davis-Richardson and Triplett, 2015). Meanwhile, certain *Bacteroides* species may be considered as the opportunistic pathogens in the human gut (Bloom et al., 2011). The gout-enriched *Porphyromonadaceae*, one family of opportunistic pathogens, is also found being higher in ankylosing spondylitis (Costello et al., 2015) and Crohn’s disease (Mondot et al., 2016). The gout-enriched *Rhodococcus*, is observed being higher in Crohn’s disease (Guerrero et al., 2015) and SLE disease (He et al., 2016). The other gout-enriched *Erysipelatoclostridium* and *Anaerolineaceae* are also considered as human opportunistic pathogens in the human gut (Sibley et al., 2012; Woting et al., 2014). Summary, the up-regulation of opportunistic pathogens may be the signatures of gout disease.

**FIGURE 4:**
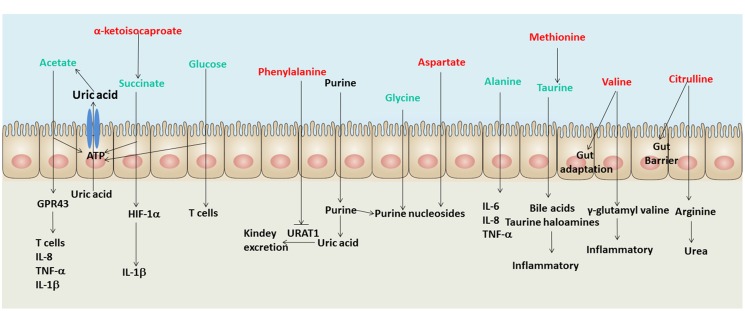
**A schematic diagram showing the main functions of the significantly altered fecal metabolites.** Red text denotes depletion in gout patients; green text denotes enrichment in gout patients. Uric acid (UA) and purine are not shown in the list of identified metabolites in the intestine, but both of them are involved in gout disease.

The up-regulation of opportunistic pathogens in the human gut may disturb host’s physical function. The metabolites in the human gut can be the intermediary of interchange between gut microbiota and human host. Therefore, this study reveals some metabolites in the human gut are associated with the discrepant microbial taxa. As shown in **Figure 4**, the signatures of metabolome in male gout patients involve multiple biological processes during the course of gout, such as UA excretion, purine metabolic disorder, and inflammatory responses.

Three gout-enriched metabolites involving energy production (acetate, succinate and glucose) may provide ATP for intestinal epithelial cells to excrete UA through the ATP-binding cassette superfamily G member 2 (Hosomi et al., 2012) and the solute carrier protein 2 family member 9 in male gout patients (Vitart et al., 2008). The gout-depleted phenylalanine has been reported as an inhibitor of URAT1 which plays a crucial role in regulating serum UA levels (Tan et al., 2014). Meanwhile, the gout-enriched glycine and gout-depleted aspartate may involve purine nucleoside biosynthesis, which induces disorders of purine metabolism (Ishikawa et al., 2013; Patel et al., 2016). Briefly, the discrepant fecal metabolites in gout patients may involve UA excretion and purine metabolism.

The gout disease is not only a metabolic disease but also an autoimmune disease. Hence, the disorders of inflammation exist in gout patients. Some signatures of metabolome in male gout patients have been reported being involving in inflammation, such as acetate that regulates T cells, IL-8, TNF-α and 1L-1β through binding GPR43 (Vieira et al., 2015), succinate that induces IL-1β through HIF-1α (Tannahill et al., 2013) and glucose that regulates T cell activation (Jacobs et al., 2008). The gout-enriched taurine in gout patients can be absorbed and metabolized into bile acids and taurine haloamines in the intestine, which both play significant roles in inflammation (Marcinkiewicz and Kontny, 2014). The gout-enriched alanine regulates the expression of inflammation factors, such as IL-6, IL-8, and TNF-α (Raspé et al., 2013). Moreover, the gout-depleted valine and citrulline may reduce the adaptation and barrier of the intestine in gout patients (Takada et al., 2006; Batista et al., 2012).

## Conclusion

The gut microbiome of gout is altered in bacterial taxa with the enrichment of several opportunistic pathogens. Most altered gut bacteria in gout has been reported being exhibited up-regulation in other autoimmune diseases. Simultaneously, the altered metabolites of gout may involve disorders of inflammation, purine metabolism, and UA excretion. The signatures of gout in fecal microbiome and metabolome may indicate potential factors of gout development.

## Compliance With Human Studies And Ethical Standards

All procedures performed in studies involving human participants were in accordance with the ethical standards of the institutional, national research committee and with the 1964 Helsinki declaration and its later amendments. Informed consent was obtained from all individual participants included in the study.

## Availability of Data and Material

The data of 26 gout patients and 26 healthy controls have been submitted to NCBI Project under accession number PRJNA359624 with NCBI Sequence Read Archive under accession number SRP096012.

## Author Contributions

All authors were involved in drafting the article or revising it critically for important intellectual content, and all authors approved the final version to be published. CW and ZH had full access to all of the data in the study and take responsibility for the integrity of the data and the accuracy of the data analysis. Study conception and design: CW and TS. Acquisition of data: ZH and LS. Analysis and interpretation of data: TS, HL, ZX, and ZH.

## Conflict of Interest Statement

The authors declare that the research was conducted in the absence of any commercial or financial relationships that could be construed as a potential conflict of interest.

## Footnotes

1 http://www.hmdb.ca/
