# Supplementary material for: Combined Signature of the Fecal Microbiome and Metabolome in Patients with Gout
Source: Front Microbiol. 2017 Feb 21;8:268. doi: 10.3389/fmicb.2017.00268 (PMC5318445; doi:10.3389/fmicb.2017.00268)
Supplement: Supplementary file 1 [file Table_1.PDF]

Table S1. List of the metabolites found in the contents of extracts of feces [a]

N.	Metabolites	Moieties	δ ^{1}H (ppm) and multiplicity
1	butyrate	CH_3,βCH_2,αCH_2	0.90(t),1.56(m),2.15(t)
2	α-ketoisocaproate	2*CH_3,CH,CH_2	0.92(d),2.06(m),2.61(d)
3	isoleucine	δCH_3,γCH_3,γCH_2,γ'CH_2,βCH,αCH	0.94(t),1.01(d),1.25(m),1.48(m),1.98(m),3.67(d)
4	leucine	δCH_3,δCH_3,γCH,βCH_2,αCH	0.96(d),0.97(d),1.69(m),1.71(m),3.74(t)
5	valine	γCH_3,γCH_3,βCH,αCH	0.99(d),1.04(d),2.27(m),3.62(d)
6	propionate	CH_3,CH_2	1.06(t),2.19(q)
7	ethanol	CH_3, CH_2	1.19(t),3.67(q)
8	lactate	βCH_3,αCH	1.33(d),4.11(q)
9	threonine	γCH_3,αCH,βCH	1.33(d),3.59(d),4.26(m)
10	alanine	βCH_3,αCH	1.48(d),3.79(q)
11	lysine	γCH_2,δCH_2,βCH_2,εCH_2,αCH	1.48(m),1.72(m),1.90(m),3.03(t),3.76(t)
12	citrulline	γCH_2,βCH_2,δCH_2,αCH	1.57(m),1.87(m),3.15(t),3.75(t)
13	arginine	γCH_2,βCH_2,δCH_2,αCH	1.73(m),1.93(m),3.03(t),3.75(t)
14	acetate	CH_3	1.92(s)
15	proline	γCH_2,βCH_2,β'CH_2,δCH_2,δ'CH_2,αCH	2.01(m),2.07(m),2.36(m),3.34(m),3.45(m),4.13(m)
16	methionine	δCH_3,βCH_2,γCH_2,αCH	2.14(s),2.16(m),2.65(m),3.86(m)
17	glutamate	βCH_2,β'CH_2,γCH_2,αCH	2.10(m), 2.09(m),2.36(m),3.77(m)
18	succinate	2*CH_2	2.41(s)
19	glutamine	βCH_2,γCH_2,αCH	2.10(m),2.46(m),3.77(m)
20	aspartate	βCH_2,β'CH_2,αCH	2.68(m),2.82(m),3.91(m)
21	asparagine	βCH_2,β'CH_2,αCH	2.86(dd),2.96(dd),4.00(m)
22	trimethylamine	3*CH_3	2.88(s)
23	dimethylglycine	CH_3	2.76(s)
24	creatine	CH_3,CH_2	3.04(s),3.93(s)
25	choline	$N(CH_3)_3$,NCH_2,OCH_2	3.21(s),3.52(m),4.07(m)
26	taurine	CH_2SO_3,NCH_2	3.25(t),3.43(t)
27	methanol	CH_3	3.37(s)
28	glycine	CH_2	3.57(s)
29	β-glucose	2CH,4CH,5CH,3CH,CH_2C_6,1CH	3.28(dd),3.41(t),3.47(dd),3.50(t),3.91(dd),4.65(d)
30	α-glucose	2CH,4CH,3CH,CH_2C_6,CH_2C_6',5CH,1CH	3.53(dd),3.42(t),3.71(t),3.74(m),3.84(m),3.84(dd),5.23(d)
31	UDP-glucose	CH	5.62(m)
32	uracil	CH,CH	5.81(d),7.54(d)
33	fumarate	2*CH	6.53(s)
34	tyrosine	βCH_2,β'CH_2,αCH,3 or 5CH,2 or 6CH	3.06(dd),3.20(dd),3.94(dd),6.91(d),7.20(d)
35	histidine	βCH_2,β'CH_2,αCH,5CH,3CH	3.14(dd),3.25(dd),3.99(dd),7.08(s),7.83(s)
36	phenylalanine	βCH_2,β'CH_2,αCH,2 or 6CH, 4CH,3	3.13(dd),3.29(dd),3.98(dd),7.33(m),7.38(m)

		or 5CH	,7.43(m)
37	tryptophan	βCH_2,$\beta' CH_2$,αCH,5CH,6CH,<br>2CH,7CH,4CH	3.31(dd),3.49(dd),4.06(dd),7.21(t),7.29(t),<br>7.33(s),7.55(d),7.74(d),
38	urocanate	CHCOOH,CH(ring),5CH,3CH	6.40(d),7.31(d),7.43(s),7.89(s)
39	hypoxanthine	CH, CH	8.20(s), 8.21(s)
40	formate	CH	8.45(s)
41	inosine	CH_2,'CH_2,5H',4H', 2H', 8H,2H	3.85(dd),3.92(dd),4.28(q),4.44(t),6.10(d),<br>8.24(s),8.34(s)
42	bile acids	C18 axial methyl	0.70(m)
43	β-xylose	1CH,2CH,3CH	4.58(d),3.24(dd),3.47(t)
44	β-arabinose	1CH,2CH,3CH	4.52(d),3.52(dd),3.69(m)
45	α-arabinose	1CH,2CH	5.21(d),3.87(d)
46	α-xylose	1CH,2CH,3CH	5.20(d),3.53(dd),3.68(m)

[a] s, singlet; d, double; t, triplet; q, quartet; m, multiplet; dd, double of doubles.
