# Supplementary figures and images for: Combined Signature of the Fecal Microbiome and Metabolome in Patients with Gout
Source: Front Microbiol. 2017 Feb 21;8:268. doi: 10.3389/fmicb.2017.00268 (PMC5318445; doi:10.3389/fmicb.2017.00268)

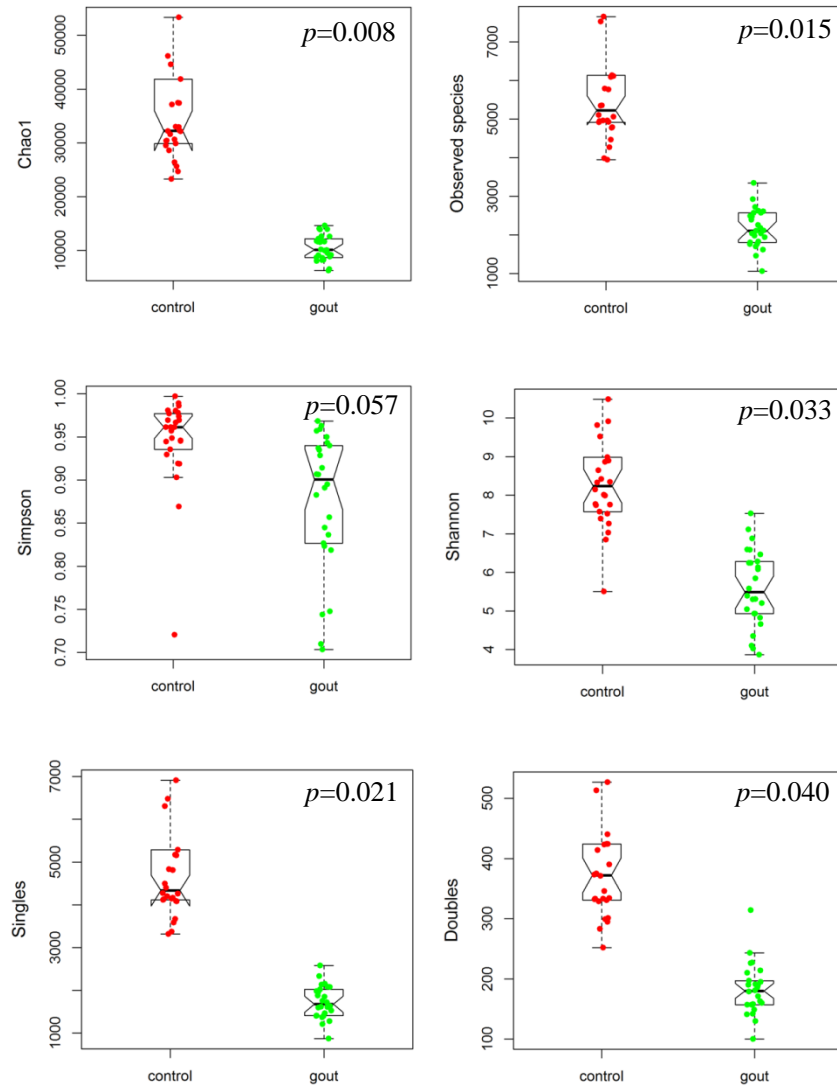

**Figure S1** The alpha-diversity indexes between healthy controls and gout patients samples.

Supplement: Supplementary file 2 [file Image_1.PDF]
