# Supplementary material for: Combined Signature of the Fecal Microbiome and Metabolome in Patients with Gout
Source: Front Microbiol. 2017 Feb 21;8:268. doi: 10.3389/fmicb.2017.00268 (PMC5318445; doi:10.3389/fmicb.2017.00268)
Supplement: Supplementary file 3 [file Image_2.PDF]

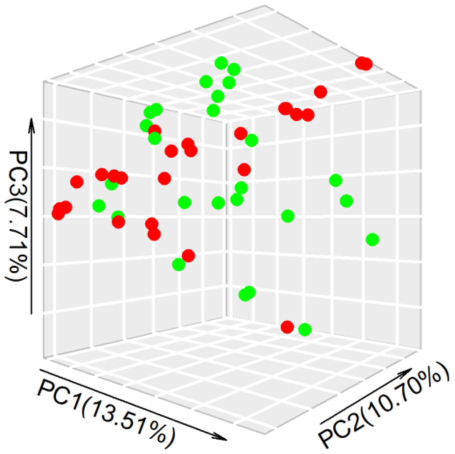

Figure S2 PCoA score plots of gout patients and healthy controls based on the gut microbial composition. Red and green represent healthy controls and gout samples, respectively. The PERMANOVA result shows *p* value based on beta-diversity of gut microbiome is 0.001 between healthy controls and gout samples.
