# Supplementary material for: Combined Signature of the Fecal Microbiome and Metabolome in Patients with Gout
Source: Front Microbiol. 2017 Feb 21;8:268. doi: 10.3389/fmicb.2017.00268 (PMC5318445; doi:10.3389/fmicb.2017.00268)
Supplement: Supplementary file 4 [file Image_3.PDF]

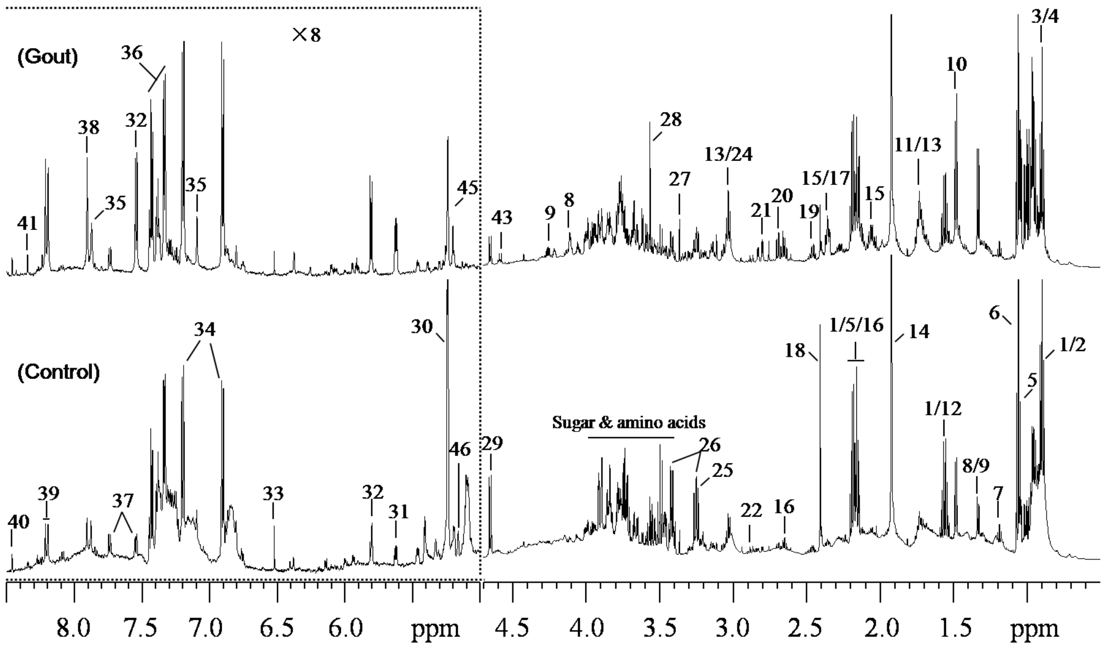

Figure S3 Typical 600M ^{1}H NMR spectra of fecal extracts for a patient with gout, and a healthy individual. The spectra in the region (δ5.0-8.5) were vertically expanded 8 times compared with the region (δ0.5-4.7). 1. Butyrate; 2. α-ketoisocaproate; 3. Isoleucine; 4. Leucine; 5. Valine; 6. Propionate; 7. Ethanol; 8. Lactate; 9. Threonine; 10. Alanine; 11. Lysine; 12. Citrulline; 13. Arginine; 14. Acetate; 15. Proline; 16. Methionine; 17. Glutamate; 18. Succinate; 19. Glutamine; 20. Aspartate; 21. Asparagines; 22. Trimethylamine; 23. Dimethylglycine; 24. Creatine; 25. Choline; 26. Taurine; 27. Methanol; 287. Glycine; 29. β-glucose; 30. α-glucose; 31. UDP-glucose; 32. Uracil; 33. Fumarate; 34. Tyrosine; 35. Histidine; 36. Phenylalanine; 37. Tryptophan; 38. urocanate; 39. Hypoxanthine; 40. Formate; 41. Inosine; 42. Bile acids; 43. β-xylose; 44. β-arabinose; 45. α-arabinose; 46. α-xylose
