# Supplementary material for: Combined Signature of the Fecal Microbiome and Metabolome in Patients with Gout
Source: Front Microbiol. 2017 Feb 21;8:268. doi: 10.3389/fmicb.2017.00268 (PMC5318445; doi:10.3389/fmicb.2017.00268)
Supplement: Supplementary file 5 [file Image_4.PDF]

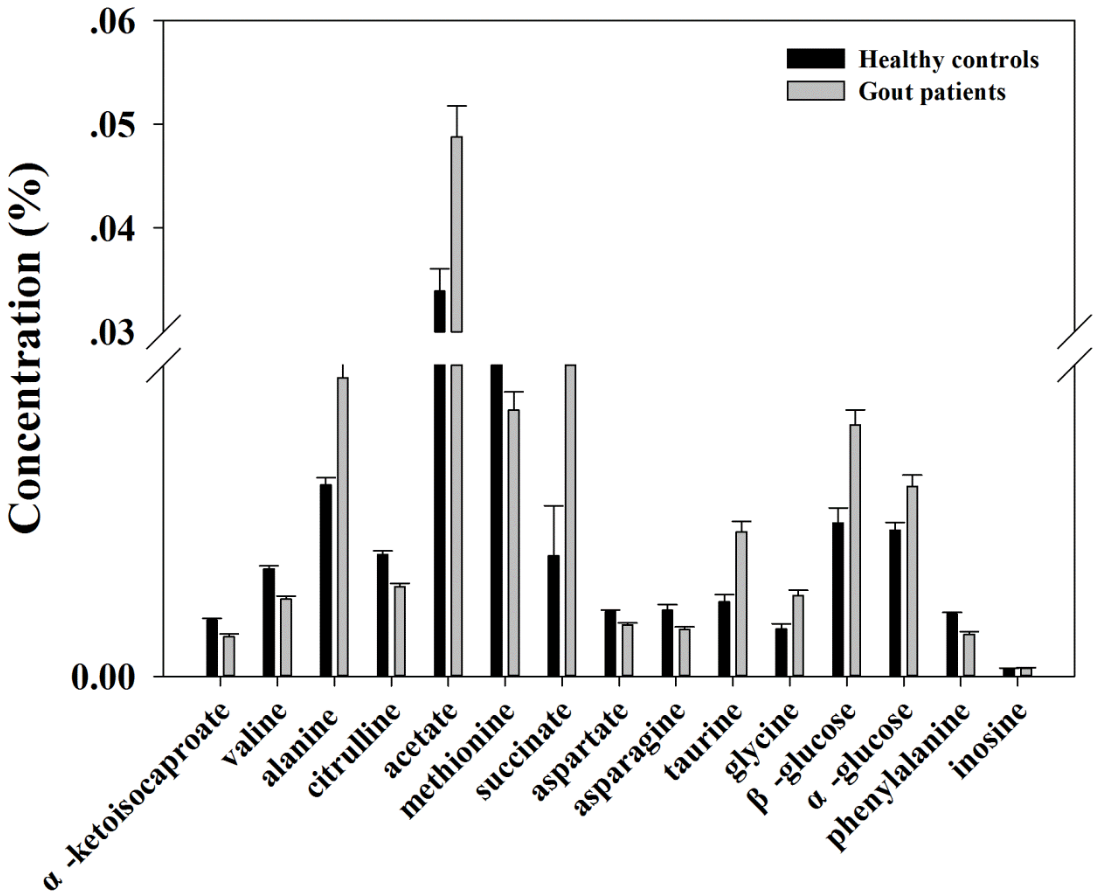

Figure S4 The detailed concentration of significantly altered metabolites between healthy controls and gout patients.
